# The Association of RAS Association Domain Family Protein1A (RASSF1A) Methylation States and Bladder Cancer Risk: A Systematic Review and Meta-Analysis

**DOI:** 10.1371/journal.pone.0048300

**Published:** 2012-11-06

**Authors:** Tianyi Gao, Shukui Wang, Bangshun He, Yuqin Pan, Guoqi Song, Ling Gu, Liping Chen, Zhenling Nie, Yeqiong Xu, Rui Li

**Affiliations:** 1 Central Laboratory, Nanjing First Hospital, Nanjing Medical, University, Nanjing, Jiangsu, People’s Republic of China; 2 Department of Life Sciences, Nanjing Normal University, Nanjing, Jiangsu, People’s Republic of China; University of North Carolina School of Medicine, United States of America

## Abstract

RAS association domain family protein 1a (RASSF1A) is a putative tumor suppressor gene located on 3p21, has been regarded playing important roles in the regulation of different types of human tumors. Previous reports demonstrated that the frequency of RASSF1A methylation was significantly higher in patients group compared with controls, but the relationship between RASSF1A promoter methylation and pathological features or the tumor grade of bladder cancer remains controversial. Therefore, A meta-analysis of published studies investigating the effects of RASSF1A methylation status in bladder cancer occurrence and association with both pTNM (p, pathologic stage; T, tumor size; N, node status; M, metastatic status) and tumor grade in bladder cancer was performed in the study. A total of 10 eligible studies involving 543 cases and 217 controls were included in the pooled analyses. Under the fixed-effects model, the OR of RASSF1A methylation in bladder cancer patients, compared to non-cancer controls, was 8. 40 with 95%CI = 4. 96–14. 23. The pooled OR with the random-effects model of pTNM and tumor grade in RASSF1A methylated patients, compared to unmethylated patients, was 0. 75 (95%CI = 0. 28–1. 99) and 0. 39 (95%CI = 0. 14–1. 09). This study showed that RASSF1A methylation appears to be an independent prognostic factor for bladder cancer. The present findings also require confirmation through adequately designed prospective studies.

## Introduction

Bladder cancer is the most common malignancy of the urinary tract, and the 9th most common cancer diagnosis worldwide, with more than 330, 000 new cases each year and more than 130, 000 deaths per year. It's generally estimated that male:female incidence ratio is 3. 8∶1. 0 [1]. The histological and pathological type of bladder cancer is mainly urothelial carcinoma, also called transitional cell carcinoma, accounting for approximately 90% [2]. Other types including squamous cell carcinoma and adenocarcinoma, account for 3–7% and <2% respectively [3].

DNA methylation of the promoter regions is emerging as the major mechanism of inactivation of TSGs (tumor suppressor gene). DNA is methylated only at cytosines located 5' to guanosines in CpG dinucleotides and DNA methylation is a frequent epigenetic event in many human cancers [4]. This modification has important regula-tory effects on gene expression, especially when involving CpG-rich areas known as CpG islands, located in the promoter regions of many genes. In many cases, aberrant methylation of the CpG island genes has been correlated with a loss of gene expression, and it is proposed that DNA methylation provides an alternate pathway to gene deletion or mutation for the loss of TSG function. Markers for aberrant methylation may represent a promising method for monitoring the occurance and progression of cancer. The RASSF1 (Ras-association domain family 1) family of proteins represents a class of Ras effectors that possess tumor suppressive properties. RASSF1A, one of the seven different isoforms of RASSF1, is a putative tumor suppressor gene located on 3p21, a region of common heterozygous and homozygous deletions in different types of human tumors [5]–[6]. It shares high sequence homology with a known mouse protein (Nore1) and may serve as an effector that mediates the apoptotic effects by binding Ras in a guanosine triphosphate-dependent manner [6]. Subsequent reports indicate that hypermethylation of the CpG islands within the RASSF1A promoter region, rather than classic mutation/deletion events, are the major cause of loss-of-expression [6]. Cells treated with demethylation agents re-express RASSF1A confirming the role of DNA methylation in the inactivation of RASSF1A in tumor cell lines [7]. In many human solid organ tumors, methylation of RASSF1 has been identified [Genebank Accession # AC002481, nucleotides 17730–18370] and its frequency varies between 30% and 50% [8], [9]. Furthermore, RASSF1A methylation was reported as a prognostic indicator in renal cell carcinoma, non-small cell lung cancer, neuroblastoma, melanoma, endometrial cancer and breast cancer [10]–[17]. All of these findings suggested that it might play a pivotal role in the development of human cancer.

**Table 1:** Characteristics of studies included in this meta-analysis.

First author	Year	Location	Patient and control	Method	RASSF1A(M/U)^b^		pTNM^a^(M/U)^b^		Grade(M/U)^b^	
					case	control	≤ T1	≥ T2	Low-grade	High-grade
Pi-Che Chen	2011	Taiwan	Tissue samples from 104 bladder UC patients and paired voided urinefrom 30 patients were collected (mdian age 70. 5years, range 40–92). 19 urinesamples from Age-and sex-matched non-cancer controls	QMSP	9/21	3/16	26/56	16/6	5/29	37/33
Reza R.	2011	Denmark	tumor tissue from 105 patients and voided urine samples from 101paired urine samples. median age 70. 2 years (range 39–91)Urine samples werealso collected from 33 control patients (median age67. 7 years, range 30–91 years)	QMSP	18/83	0/33	25/61	6/13	-	-
Hui-Hui Lin	2010	Taiwan	tissues and 100 ml preoperative urine were sampled from 57 patients.(median age 64, range 39–90) Urine specimens were also taken from 20 healthycontrols.	MSP	37/20	0/20	25/7	17/8	16/3	26/12
Priscilla D Negraes	2008	Brazil	39 archived tumor fragments and 23 washouts from bladder washingsof patients (median age of 67. 85 years, ranging from 40 to 90years) and acontrol group included 24 urinary bladder washings from patients withoutany bladder tumor history	MSP	8/8	5/19	-	-	3/18	8/20
Sonata Jarmalaite	2008	Finland	Tumor tissues from 58 bladder cancer patients with a mean age of66 years (range: 37–85) and 3 healthy control tissues	MSP	36/22	0/5	11/27	10/10	1/9	16/22
jian yu	2007	china	urine sediments from 132 bladder cancer patients, 23 age-matchedpatients with noncancerous urinary lesions, 6 neurologic diseases,and 7 healthy volunteers.	MSP	47/85	2/34	-	-	-	-
DR Yates	2006	UK	Urine samples were obtained prospectively from 35 UC patients with a new diagnosis of UC had a median age of 75 years (range 54–92) and 34 healthy volunteers under the age of 40 years	QMSP	18/17	9/25	-	-	-	-
Essel Dulaimi,	2004	USA	tumor tissue and urine from 45 patients (age, 37–85 years) Urine control specimens from 12 normal, healthy individuals and 9 patients with earlyurinary disease	MSP	18/27	0/21	15/7	8/9	2/2	21/20
Michael W. Y	2003	Hong Kong	40 bladder tumor tissues samples and 14 urine samples were collected from patients had a median age of 70 years (range 47–87) and 10 normal voidedurine sediments from age- and gender-match control	MSP	7/7	0/10	7/29	10/10	1/9	16/14
Min-Goo Lee	2001	Korea	Fifty-five primary bladder carcinomas and 15 age- and gender-match normal bladder tissues were obtained from 55 bladder cancer patients and15 noncancer patients	MSP	31/24	0/15	-	-	-	-

MSP, methylati on specific PCR; QMSP, quantitative methylation specific PCR. pTNM (p, pathologic stage; T, tumor size; N, node status; M, metastatic status) Tumor grade ≤1 was defined as low-grade, and tumor grade ≥2 was defined as high-grade.

a p, pathologic stage; T, tumor size; N, node status; M, metastatic status;

b RASSF1A methylated/RASSF1A unmethylated.

Despite a number of individual studies performed in bladder cancer patients, the prognostic value of RASSF1A methylation status in bladder cancer patient’s diagnosis and the relationship between RASSF1A methylation and pathological features or the tumor grade of bladder cancer remains controversial. Therefore, a systematic review was performed of the literature with meta-analysis to obtain a more accurate evaluation of its prognostic value in bladder cancer.

**Table 2:** Stratified analyses of RASSF1A methylation and bladder cancer risk.

Variables	p^a^	OR	95% CI	Heterogeneity		
				X^2^	P	I^2^
RASSF1A						
total	10	8. 40	4. 96–14. 23	13. 35	0. 15	32. 6%
material						
Urine	8	7. 29	4. 20–12. 65	10. 65	0. 15	34. 3%
Tissue	2	28. 76	3. 73–221. 59	0. 15	0. 70	0. 0%^a^
method						
QMSP	3	3. 68	1. 69–8. 03	1. 53	0. 47	0. 0%^a^
MSP	7	14. 76	6. 89–31. 61	6. 01	0. 42	0. 2%

a Number of comparisons.

b Between group heterogeneity not calculated; only valid with inverse variance method.

**Table 3:** Main results of eligible studies evaluating RASSF1A methylation and pTNM/grade in bladder cancer.

Variables	p^a^	OR	95% CI	Heterogeneity		
				X^2^	P	I^2^
pTNM	6	0. 75	0. 28–1. 99	20. 54	0. 001	75. 7%
Grade	6	0. 39	0. 14–1. 09	11. 81	0. 037	57. 7%

a Number of comparisons.

## Materials and Methods

### Publication Selection

Studies were identified via an electronic search of PubMed and EMBASE using the following key words: bladder cancer, UBC, RAS association domain family protein1A, RASSF1A, methylation, prognostic, prognosis, pathological features and tumor grade. We also manually searched the references of these publications in order to retrieve additional studies. Only those published as full-text articles and in English were included as candidates. The search updated on 28 July 2012.

**Figure 1:**
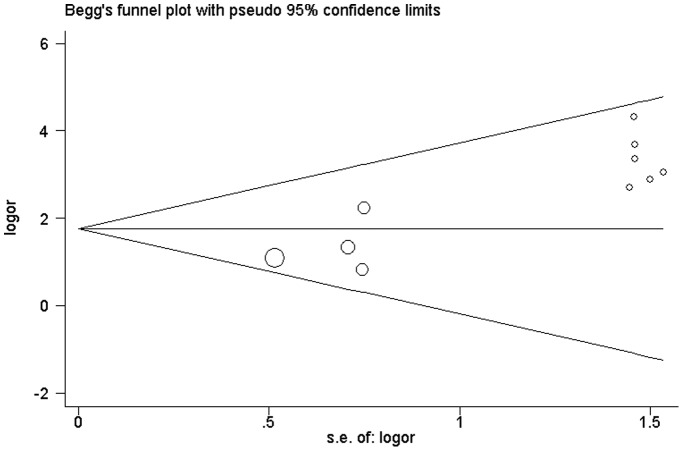
Begg's funnel plot with pseudo 95% confidence limits of publication bias test for RASSF1A methylation. Each point represented a separate study for the indicated association. Logor natural logarithm of OR, horizontal line mean effect size. Fig. 1: Begg’s funnel plot of publication bias test.

**Figure 2:**
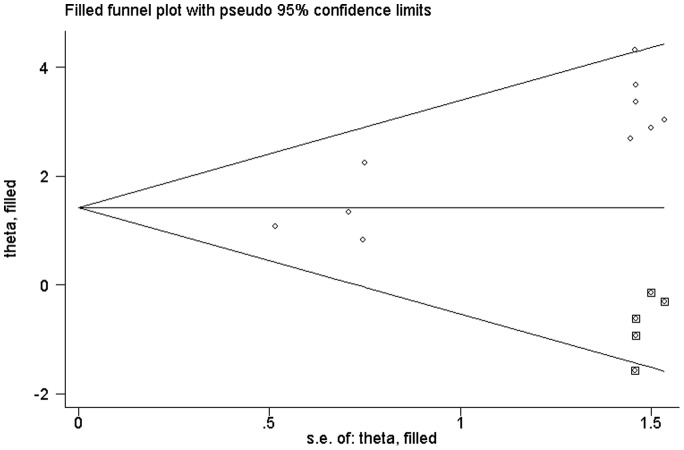
Begg’s funnel plot of publication bias test after trim-and-fill method.

### Inclusion and Exclusion Criteria

Studies were selected for analysis if they met the following criteria: 1) they were original epidemiological studies on the correlation between RASSF1A promoter methylation and the prognosis of bladder cancer patients, pathological features or the tumor grade of bladder cancer; 2) RASSF1A methylation status was examined using methylation-specific PCR (MSP) or quantitative MSP (QMSP); 3) the subjects in every study comprised patients and non-cancer controls; 3) studies should be with full text not only abstracts for relevant information extraction; 4) when the same patient population reported in several publications, only the most recent report or the most complete one was included in this analysis to avoid overlapping between cohorts; 5) the numbers of patients and controls in each study should be more than 3 respectively.

### Data Collection

For each eligible study, we collected information regarding authors, year and source of publication, country of origin, inclusion criteria, exclusion criteria, pathological features, tumor grade, RASSF1A methylation frequencies in non-cancer controls and patients of bladder cancer and the method for methylation detection. All included studies used non-cancer people as a control group, though some of them did not provide the definition of noncancer. In studies defining non-cancer people, there are two definitions: (1) normal healthy person; (2) people with urological disease but no prior history of genitourinary malignancy. Since it is impossible to redefine non-cancer people on a unified standard, we combined non-cancer people in our meta-analysis according to their original group in each individual study. Of these studies, tumor grade ≤1 was defined as low-grade, and tumor grade ≥2 was defined as high-grade which were defined by cellular differentiation. The final eligible articles selected for further meta-analysis were evaluated independently by two reviewers. Minor discrepancies were resolved by the authors' discussion.

### Meta-analysis and Statistical Analysis

The foremost analysis examined the differences in the frequency of RASSF1A methylation between bladder cancer patients and non-cancer people by odds ratio (OR) with the corresponding 95% CI. Moreover, the strength of association between RASSF1A methylation and patients' pTNM (p, pathologic stage; T, tumor size; N, node status; M, metastatic status) and tumor grade were also assessed by OR with the corresponding 95% CI. To assess heterogeneity across the studies, a statistical test for heterogeneity was performed based on the statistics [18]. If the studies were shown to be homogeneous with P>0. 05 for the Q-statistics, the summary of OR was calculated by a fixed-effects model (the Mantel-Haenszel method) when between-study heterogeneity was absent [19]. Otherwise, a random-effects model (the DerSimonian and Laird method) was selected [20]. In addition, stratified analyses were also performed by material and method. furthermore, a sensitivity analysis, by which a single study in the meta-analysis was deleted each time to determine the influence of the individual data set to the overall pooled OR, was performed to assess the stability of the results. The potential publication bias was examined visually in a funnel plot of log [OR] against its standard error (SE), and the degree of asymmetry was tested by Egger's test [21]. This meta-analysis was performed using the software STATA version 12. 0. All P-values were based on two-sided tests and a P-value of less than 0. 05 was considered statistically significant.

## Results

### Study Characteristics

According to our inclusion criteria, a total of 10 eligible studies[22]–[31] involving 543 cases and 217 controls were included in the pooled analyses. The characteristics of these studies are summarized in Table 1. Of these studies, six studies were conducted in Asia, two were in Europe, and the rest were in USA, Brazil. The methylated RASSF1A levels were detected using either methylation specific PCR (MSP)[22], [24], [25], [28]–[31] or quantitative methylation specific PCR (QMSP) [23], [26], [27]. DNA methylation status of RASSF1A promoter was assessed in urine or tumor tissues. Bladder cancers were confirmed histologically or pathologically in all the studies.

### Meta- analysis

In general, the frequencies of RASSF1A methylation were tested in ten reliable studies. The main results were summarized in Table 2. Under the fixed-effects model, the pooled OR of RASSF1A methylation in bladder cancer patients, compared to non-cancer controls, was 8. 40 with 95%CI = 4. 96–14. 23. In the stratified analysis by material, significantly increased risks were found in urine samples in detction RASSF1A methylation in bladder cancer(OR = 7. 29, 95%CI = 4. 20–12. 65 ) and in tissues (OR = 28. 76, 95%CI = 3. 73–221. 59 ). As stratified analysis by method, significantly increased risks were also found in MSP(OR 14. 76, 95% CI = 6. 89–31. 61) and QMSP (OR = 3. 68, 95%CI = 1. 69–8. 03). In the evaluating RASSF1A methylation and pTNM/grade in bladder cancer, each was carried out in six studies. The main results were summarized in Table 3. Under the random-effects model, the pooled OR of pTNM and tumor grade in RASSF1A methylated patients, compared to unmethylated patients was 0. 75 (95%CI = 0. 28–1. 99) and 0. 39 (95%CI = 0. 14–1. 09).

### Sensitivity Analyses

Sensitivity analysis revealed that four independent studies were the main source of heterogeneity [22]–[25]. Then the heterogeneity of RASSF1A methylation in bladder cancer patients, compared to non-cancer controls was decreased when these four studies were removed (P = 0.49 ). In addition, no other single study was found to impact the pooled OR as indicated by sensitivity analyses.

### Publication Bias

As shown in Figure 1, the shape of the funnel plots seemed asymmetrical in the methylation comparison between bladder cancer patients and non-cancer controls, suggesting the presence of publication bias. Then, the Egger’s test provides statistical evidence of funnel plot asymmetry (t = 5. 14, P = 0. 001). To adjust this bias, a trim-and-fill method developed by Duval and Tweedie [32] was implemented (Figure 2). Meta-analysis with or without the trim-and-fill method did not draw different conclusions, indicating that our results were statistically robust. Funnel plot and Egger’s test were performed to assess the publication bias in studies of association between RASSF1A methylation and pTNM/grade, The shape of the funnel plot did not indicate any evidence of obvious asymmetry (figure not shown) and the Egger's test suggested the absence of publication bias (P>0. 05).

## Discussion

The results of our systematic review showed that RASSF1A methylation in bladder cancer was associated with tumor risk as either detected in urine or tissue by MSP or QMSP. However, the RASSF1A methylation was not associated with increased risk for developing pathological features or the tumor grade of bladder cancer in comparison btween RASSF1A methylated bladder cnacer patients and unmethylated patients.

Accumulated data documented that bladder cancer patients always show RASSF1A methylation [31]. Previous reports also demonstrated that genetic variations of RASSF1A affect bladder cancer susceptibility [28] and the frequency of RASSF1A methylation was found to be significantly higher in patients group compared with controls [27], [28]. To further confirm RASSF1A promoter methylation status in bladder cancer patient’s diagnosis, we carried out a meta-analysis of 10 studies involving 543 cases and 217 controls to derive a more precise estimation of the association. Our results suggested that RASSF1A methylation is a potential risk factor for bladder cancer as detected both in urine and tumor tissues. The frequency of RASSF1A methylation in bladder cancer patients was 8. 40 times higher than that in Non-cancer people. However, MSP is a nonquantitative nonfluorometric PCR method to investigate promoter methylation. This method may fail to detect low concentrations of methylated alleles, unlike QMSP which can detect up to 1/1000 methylated alleles [27]. In this meta-analysis, both of them present a positive effect in detection in RASSF1A methylation. Furthermore, frequent methylation was detected in RASSF1A with significant associations with tumor stage, grade and muscle invasiveness [30], [38] which was not found in other studies [27], [31]. To resolve the conflicting results, we also carried out a meta-analysis which indicated that the frequency of RASSF1A methylation did not correlate with the pTNM or tumor grade of bladder cancer patients. These results suggested that inactivation of RASSF1A may be an early event in bladder carcinogenesis.

Epigenetic alterations are a hallmark of human cancer. In particular, DNA methylation is a common mechanism for inactivating tumor-suppressor and other cancer genes in tumor cells [33]. The aberrant methylation patterns have been used as targets for the detection of tumor cells in clinical specimens such as tissue biopsies or body fluids [34]. RAS association domain family protein 1A (RASSF1A) is a putative tumor suppressor gene located on 3p21, has been regarded playing important roles in the regulation of different types of human tumors [5], [6]. It has been well documented that Ras proteins bind a diverse array of effector molecules and mediate tumor suppressive effects such as terminal differentiation and apoptosis as well as oncogenic effects [35], [36]. Moreover, activation of Ras signaling pathway is a major event in the process of cancer development. Mutations within the Ras proto-oncogene commonly occur in cancer, leading to its hyperactivation, aberrant growth signaling, and unchecked cell proliferation. It was suggested that RASSF1 might mediate the Ras-activated growth inhibition through its proapoptotic function and RASSF1A inactivation may be a tumorigenic mechanism that is distinct from the oncogenic activation of Ras signaling in tumors [37]. Previous studies have also demonstrated that arsenic pollution is associated with DAPK and RASSF1A methylation in bladder cancer [39], [40]. It may also be one of the factors that contribute to this distinct methylation epigenotype.

After all, this meta-analysis still exist some limitations. First, the controls included in the analysis were not uniform. Most of the controls were healthy population while some of them were patients. In this way, some of the controls, especially those who have benign disease should have different risks suffering from bladder cancer. Second, there were only two studies of detection in tissues in the subgroup analysis. The sample size was too small to have substantial power to explore the real association. Third, there were only six literatures enrolled in meta-analysis of association between methylation and pTNM/grade, and the between-study heterogeneity was observed. Therefore, the pooled ORs were calculated by the random model. Fourth, the detailed information (such as age, sex, and life-style) could not be traced so that our unadjusted estimates should be confirmed by further studies.

### Conclusion

Our meta-analysis suggested that detection of RASSF1A methylation in voided urine is a potential non-invasive diagnostic tool in bladder cancer. It is necessary to conduct large sample size studies of the association between RASSF1A methylation and bladder cancer risk, eventually leading to our better understanding.
